# Development and Psychometric Validation of the Physical Activity and Health Literacy Scale (PA‐HLS) for College Students

**DOI:** 10.1155/nrp/9980613

**Published:** 2026-05-13

**Authors:** Pei Duan, Xia Song, Xinrui Zhang, Liqian Ruan, Song Chen

**Affiliations:** ^1^ Faculty of Medicine, School of Nursing, Yangzhou University, Yangzhou, Jiangsu, China, yzu.edu.cn; ^2^ National Clinical Research Center for Kidney Diseases, Jinling Hospital, Nanjing, Jiangsu, China, nju.edu.cn

**Keywords:** exercise, health literacy, psychometrics, students, surveys and questionnaires

## Abstract

**Background:**

Declining physical fitness among college students has become a global public health concern. Existing physical literacy and health literacy instruments do not adequately capture the competencies required to access, interpret, and critically evaluate physical activity–related health information in contemporary digital environments.

**Objective:**

This study aimed to develop and psychometrically validate the Physical Activity and Health Literacy Scale (PA‐HLS) for college students based on Nutbeam’s hierarchical model of health literacy.

**Methods:**

An initial item pool was generated through a systematic literature review and refined via two rounds of Delphi expert consultation (*n* = 19) and pilot testing (*n* = 30). The final 15‐item PA‐HLS was administered to 442 Chinese college students. Psychometric evaluation included content validity assessment, item analysis, exploratory and confirmatory factor analyses, internal consistency reliability (Cronbach’s α and McDonald’s ω), discriminant validity testing, and Rasch model analysis.

**Results:**

All items demonstrated good discrimination (critical ratios = 6.74–31.90, *p* < 0.001) and strong factor loadings (0.59–0.93). Content validity was excellent (I‐CVI = 1.00; S‐CVI = 1.00). Exploratory factor analysis supported a three‐factor structure explaining 77.1% of the total variance, which was confirmed by confirmatory factor analysis with acceptable model fit. Internal consistency was high for the total scale (*α* = 0.892; *ω* = 0.869) and all subscales. Rasch analysis further supported the scale’s measurement quality.

**Conclusion:**

The PA‐HLS is a theoretically grounded and psychometrically robust instrument for assessing physical activity–related health literacy among college students and may inform targeted health promotion interventions in university settings.

## 1. Introduction

Declining physical fitness among college students has become a prominent public health issue in China and worldwide. Large‐scale epidemiological evidence indicates a substantial deterioration in multiple physical health indicators among Chinese college students over the past 2 decades. Between 2000 and 2019, the comprehensive physical fitness index declined markedly, accompanied by a sharp increase in the prevalence of overweight, obesity, and elevated blood pressure as well as a 6‐fold increase in cardiometabolic comorbidity rates [1]. Physical inactivity is prevalent in this population, with almost 50% of college students engaging in physical exercise fewer than three times per week [2]. These trends highlight a critical need for effective strategies to promote sustainable physical activity (PA) behaviors among university students.

Physical literacy (PL) is widely recognized as a key determinant of PA engagement and related health outcomes [3]. Conceptually, PL encompasses the ability to acquire, understand, evaluate, and apply information related to PA, together with motivation, confidence, and embodied competence [3]. Empirical studies have demonstrated that higher levels of PL are associated with greater participation in moderate‐to‐vigorous PA and improved physical and mental health outcomes, including cardiorespiratory fitness, body composition, and psychological well‐being [4–6].

To date, several instruments have been developed to assess PL across diverse populations. The Canadian Assessment of Physical Literacy evaluates physical competence, daily behavior, motivation, confidence, and knowledge [3, 7], while the Physical Literacy Assessment for Youth tools provide multi‐informant assessments tailored to children and adolescents [8, 9]. Other instruments [5, 7] target motor skills [9, 10], PA behaviors [4, 11, 12], or psychosocial correlates of PA. Although these tools have contributed substantially to PL measurement, most of them focused on physical competence, affective attributes, or behavioral outcomes, rather than assessing health information processing skills required to navigate contemporary PA‐related knowledge environments.

In recent years, the rapid expansion of digital media has profoundly altered how college students access exercise‐related information. Social media platforms and online fitness content offer abundant but highly heterogeneous information, often blending scientific evidence with misinformation and commercial promotion.

In this context, the ability to critically appraise PA‐related health information has become increasingly important. However, existing PL instruments do not adequately capture competencies in accessing, interpreting, and evaluating PA‐related health information, particularly within complex digital environments.

Nutbeam’s hierarchical model of health literacy provides a theoretically robust framework for addressing this gap [13, 14]. The model conceptualizes health literacy as a progression from functional health literacy (FL; basic skills for obtaining and understanding information), to interactive health literacy (IL; skills for applying information through communication and behavioral adaptation), and ultimately to critical health literacy (CL; advanced skills for critical appraisal and informed decision‐making). Unlike Cairney’s PL model, which primarily emphasizes “doing” through physical competence and activity participation, Nutbeam’s framework emphasizes “knowing” and decision‐making processes underlying health behaviors [13, 14]. Although approximately 15 general health literacy instruments have been developed for adolescents and young adults [15], their applicability to the specific domain of PA remains limited. Existing domain‐specific scales for college students—such as those assessing environmental [16], mental [17], primary care–oriented [18], sleep [19], or reproductive health literacy [20]—do not address the unique informational and decision‐making demands associated with PA. Unlike many other health domains, PA‐related information is highly commercialized, often promoted through fitness programs, dietary supplements, and online coaching services. In addition, PA information is closely related to visible bodily outcomes, which can intensify body image concerns and shape decision‐making. The PA domain is also particularly vulnerable to the spread of pseudoscientific claims (such as “spot reduction” or myths of rapid body transformation), especially across digital and social media platforms. These characteristics highlight the need for individuals not only to access and understand information but also to critically evaluate its credibility and relevance. Consequently, the critical appraisal and decision‐making skills emphasized in PA‐HLS are essential for navigating the complex landscape of PA‐related information.

In addition to conceptual limitations, many existing instruments rely on relatively narrow validation approaches, typically combining exploratory factor analysis (EFA) with Cronbach’s α to establish internal consistency. These approaches may yield incomplete evidence regarding dimensional structure and item‐level performance. Contemporary psychometric standards increasingly recommend integrating classical test theory with item response theory methods to provide more rigorous validation [21].

Given this background, in the present study, we conceptualized Physical Activity and Health Literacy (PA‐HL) as a domain‐specific form of health literacy that links health cognition with behavioral transformation in the context of PA. Guided by Nutbeam’s three‐level model, this study aimed to develop and psychometrically validate the Physical Activity and Health Literacy Scale (PA‐HLS) for college students. By operationalizing FL, IL, and CL competencies specific to PA, the PA‐HLS seeks to extend beyond traditional PL frameworks centered on physical capability and instead focus on health information competence as a key driver of informed PA‐related decision‐making. The scale is intended to provide a theoretically grounded and methodologically robust tool for identifying PA‐related health literacy gaps and supporting targeted health promotion interventions in university settings.

## 2. Materials and Methods

### 2.1. Initial Scale Development

#### 2.1.1. Theoretical Framework

The PA‐HLS was developed based on Nutbeam’s hierarchical model of health literacy [13, 14], which conceptualizes health literacy as three progressive levels: FL, IL, and CL. FL refers to the basic ability to access and understand essential PA‐related health information. In the context of PA, this includes recognizing key exercise terminology (e.g., aerobic exercise and target heart rate), identifying potential health risks, and locating reliable sources of information. IL reflects the ability to apply health information through communication and behavioral adaptation. For PA, this involves regulating exercise intensity, managing minor injuries (e.g., applying the RICE principle), and integrating exercise habits into daily routines. CL represents advanced cognitive skills for critically evaluating health‐related information and using it to make informed decisions. This includes differentiating scientific evidence from pseudoscientific claims (e.g., “spot reduction” myths), comparing different exercise approaches, and understanding the long‐term value of health‐promoting behaviors. Within this framework, the three dimensions correspond to a progression from knowledge acquisition to behavioral application and informed decision‐making in PA. FL provides the cognitive foundation for participation, IL supports behavioral regulation, and CL enables resistance to misinformation and effective self‐management in increasingly complex health information environments (Appendix Figure A1).

#### 2.1.2. Item Pool Generation

An initial item pool was developed through a systematic literature review conducted between September and October 2024. Four electronic databases (PubMed, Web of Science, CNKI, and WanFang Data) were searched using combinations of Chinese and English keywords, including “college student,” “undergraduate,” “physical health literacy,” “exercise health literacy,” and “physical activity literacy.” All records published up to September 2024 were considered.

After duplicate removal and title–abstract screening, 48 articles were identified for full‐text review. Following detailed assessment, studies with incomplete methodological descriptions or limited relevance were excluded, resulting in 24 core articles that informed item generation. From these sources, 112 candidate items were extracted and cataloged using a structured “dimension–description–source” framework. Redundant and semantically overlapping items were consolidated, and overly technical terminologies were simplified to enhance clarity and applicability to college students.

The remaining items were preliminarily organized according to a “knowledge–skills–behavior” taxonomy aligned with Nutbeam’s health literacy framework, yielding 45 candidate items across three hypothesized dimensions. To further refine the item pool, an expert panel meeting was convened, which comprised seven specialists (one professor of sports science, four public health researchers, and two educational administrators). The panel reviewed the proposed dimensional structure and rated the relevance and clarity of each item by using a five‐point Likert scale. Based on a predefined criterion (mean importance score ≥ 3.5), 25 items were removed, resulting in 20 items retained for subsequent validation. The panel also recommended a list of experts to participate in the subsequent two‐round Delphi consultation.

#### 2.1.3. Expert Consultation (Delphi Method)

##### 2.1.3.1. Expert Selection

Experts were recruited using predefined criteria to ensure adequate methodological and substantive expertise. Eligible experts were required to have at least 5 years of professional experience in college student PA, health promotion, sports rehabilitation, or related fields as well as prior experience or familiarity with scale development. All experts held at least a bachelor’s degree and an intermediate or higher professional title.

##### 2.1.3.2. Consultation Questionnaire Development and Item Screening

A structured Delphi questionnaire was developed to evaluate the relevance and clarity of the candidate items. The questionnaire included an introductory section outlining the study objectives and instructions, followed by the core item evaluation section in which experts rated the importance of each item using a five‐point Likert scale (1 = very unimportant to 5 = very important). Open‐ended fields were provided to allow experts to suggest item deletion, addition, or modification. Additional sections collected demographic information and assessed experts’ self‐reported familiarity with the topic and the basis of their judgments (e.g., theoretical knowledge, practical experience, or literature review).

The Delphi consultation was conducted in two rounds between November and December 2024. Questionnaires were distributed electronically by email and WeChat to 20 purposively selected experts who met the inclusion criteria. Responses from the first round were summarized and used to refine item wording and content prior to the second round for achieving consensus on item retention and modification.

### 2.2. Pilot Testing

Prior to the formal survey, a pilot test was conducted with 30 college students to evaluate the clarity, comprehensibility, and feasibility of the preliminary PA‐HLS. Eligible participants were full‐time undergraduates aged 18–25 years who provided informed consent and had no severe cognitive impairments or physical conditions that could interfere with their understanding of PA‐related items. To minimize potential bias associated with specialized knowledge, students majoring in physical education or sports training and those who had participated in PA‐related research or intervention programs within the previous 3 months were excluded. Students experiencing acute injuries or illnesses at the time of data collection were also excluded. Feedback from the pilot test indicated that the overall structure and content of the scale were acceptable. Minor wording revisions were made to three items to improve clarity and ease of understanding, and no substantial changes to item content or dimensional structure were required.

### 2.3. Formal Scale Testing

#### 2.3.1. Participants

A convenience sampling strategy was used to recruit college students in China between March and May 2025. The inclusion and exclusion criteria were consistent with those applied in the pilot study. According to recommendations for parametric item response theory analyses, a minimum sample size of 200 is considered adequate [21]. The final sample comprised 442 participants, exceeding the commonly recommended ratio of at least 10 participants per item for scale validation studies. The study protocol was approved by the Ethics Committee of Yangzhou University (Approval No. YZUHL20250050), and all participants provided informed consent prior to participation.

#### 2.3.2. Instruments

A demographic variable checklist was used to collect basic participant information, including gender, age, academic major, and year of study. The PA‐HLS is a self‐administered instrument comprising 15 items across three dimensions: FL, IL, and CL. Items are rated on a four‐point Likert scale, with total scores ranging from 15 to 60. Higher scores indicate higher levels of PA‐related health literacy.

#### 2.3.3. Data Collection and Quality Control

All survey administrators received standardized training prior to data collection. Surveys were administered primarily in classroom settings using group‐based procedures. Participants completed the demographic questionnaire followed by the PA‐HLS, with an average completion time of approximately 10 min. For participants who were unavailable for in‐person data collection, an online version of the questionnaire was administered using the Wenjuanxing platform. Measures were implemented to prevent duplicate responses and ensure data quality, including restrictions on repeated submissions.

#### 2.3.4. Validity and Reliability Testing Based on Classical Test Theory [22–24]

Within the framework of classical test theory, item analysis, reliability, and validity of the PA‐HLS were systematically evaluated. Item discrimination was examined using the critical ratio (CR) method, with CR values greater than 3.0 and *p* < 0.05 indicating adequate discrimination. Item–total correlations were calculated using Pearson’s correlation coefficients; items with a correlation coefficient of < 0.40 were considered potentially weak indicators of the construct. Internal consistency reliability was assessed using Cronbach’s α and split‐half reliability, with values above 0.80 and 0.70, respectively, considered acceptable. In addition, McDonald’s ω was calculated to provide a more robust estimate of internal consistency reliability that does not assume tau‐equivalence. Content validity was evaluated using the Content Validity Index (CVI). A panel of 19 experts rated the relevance of each item, and item‐level (I‐CVI) and scale‐level (S‐CVI) indices were calculated. According to Lawshe’s recommendations [25], an I‐CVI value of 0.78 or higher was considered acceptable for expert panels exceeding 15 members. Construct validity was examined through exploratory and confirmatory factor analyses. For EFA, sampling adequacy was confirmed using the Kaiser–Meyer–Olkin (KMO) measure (> 0.70) and Bartlett’s test of sphericity (*p* < 0.05). Principal component analysis (PCA) with varimax rotation was employed to extract factors. Items with factor loadings below 0.40 or with substantial cross‐loadings (loadings > 0.40 on multiple factors with a difference of < 0.20) were removed. Confirmatory factor analysis (CFA) was subsequently conducted to validate the factor structure, and model fit was evaluated using multiple indices, including *χ* ^2^/df, RMSEA, CFI, IFI, and TLI. Discriminant validity was assessed using the Fornell–Larcker criterion [26] by comparing the square roots of the average variance extracted (AVE) for each construct with the corresponding interconstruct correlations.

#### 2.3.5. Rasch Model Analysis Based on Item Response Theory [27, 28]

Prior to the Rasch analysis, we tested the unidimensionality of each subscale—a prerequisite for applying the Rasch model. This was accomplished by PCA of residuals, wherein the first contrast eigenvalue below 3.0 supported the assumption of unidimensionality. Item fit was evaluated using both the information‐weighted mean square (Infit MNSQ) and outlier‐sensitive mean square (Outfit MNSQ) statistics. Values between 0.5 and 1.5 were considered acceptable, with values closer to 1 indicating ideal fit. The point‐measure correlation (PT‐measure CORR.) was used to assess how well each item aligned with its intended dimension; correlations between 0.4 and 0.8 were regarded as good, with 0.3 set as the minimum acceptable threshold. Standard errors were examined to gauge the stability of parameter estimates, with smaller values indicating greater precision. Person and item reliability indices were derived, with values approaching 1 indicating high measurement quality. Separation indices were used to evaluate the scale’s ability to distinguish between respondents of varying ability levels and items of differing difficulty; separation index ≥ 2 and reliability ≥ 0.7 were considered acceptable. Finally, item characteristic curves (ICCs) were generated to visually inspect the congruence between empirical data and model expectations.

### 2.4. Statistical Analysis

Data analyses were conducted using SPSS version 26.0 and AMOS version 26.0. Descriptive statistics were used to summarize participant characteristics, with continuous variables presented as mean and standard deviations ($\overset{¯}{X}\pm s$) and categorical variables reported as frequencies and percentages. During the expert consultation process, expert authority coefficients (Cr), calculated as the average of the familiarity coefficient and judgment coefficient, were used to assess expert credibility. The level of agreement among experts was evaluated using Kendall’s coefficient of concordance. For scale development and validation, item analysis included assessments of item discrimination using the CR method, item–total correlations, and factor loadings. Validity evaluation encompassed content validity, construct validity through EFA and CFA, and discriminant validity. Reliability was assessed using Cronbach’s α and split‐half reliability. Omega coefficients were computed based on standardized factor loadings derived from CFA using Python (version 3.9.13) with the pingouin package. Item response theory analysis was conducted using the Rasch model with Winsteps version 3.72 to further examine item‐level performance and unidimensionality. For all statistical analyses, a two‐tailed *p* value of < 0.05 was considered statistically significant.

## 3. Results

### 3.1. Expert Consultation Results

Nineteen experts completed two rounds of Delphi consultation, achieving a 100% valid response rate in both rounds. The expert panel represented multiple regions, including Jiangsu, Shanghai, Sichuan, and Hubei. The cohort comprised 6 males and 13 females, with a mean age of 44.84 ± 6.32 years and an average work experience of 26.68 ± 9.20 years. Educational qualifications of the cohort members included 11 bachelor’s degrees, 5 master’s degrees, and 3 PhDs. By academic rank, 15 were associate professors and 4 were full professors.

The 100% participation rate indicated strong expert engagement. The proportion of items receiving revision suggestions was 42.1% in the first round and decreased to 5.3% in the second round, indicating that a high level of consensus was reached following initial revisions. Authority coefficients (Cr) were high in both rounds (0.857 in Round 1 and 0.860 in Round 2). Kendall’s W coefficient of concordance was 0.349 (*χ* ^2^ = 146.028, *p* < 0.001) in the first round and 0.267 (*χ* ^2^ = 86.107, *p* < 0.001) in the second round, demonstrating a significant level of consensus among the experts. The mean scores for item importance improved from a range of 3.05–4.68 in Round 1 to 4.37–5.00 in Round 2, while the coefficients of variation (CV) decreased from 0.05 to 0.41 to 0.00–0.18, further confirming consensus convergence.

Based on Round 1 feedback, four items were removed due to limited applicability (e.g., FL5: “I can understand safety instructions for sports equipment”) or weak relevance to the construct (e.g., CL6/CL7/CL8 concerning public rules, environmental sustainability, and knowledge sharing). Wording revisions were implemented for 12 additional items to improve clarity and precision. Specifically, technical terminology (e.g., “supercompensation,” “RM value”) was eliminated from FL1, and the explanation of the “RICE principle” in IL2 was simplified. Redundant phrasing was removed from several CL items (CL1–CL3).

In response to Round 2 feedback, minor refinements were conducted, including removal of the term “basic” from FL1 and “bodily” from FL2. Additionally, the core content of the original item IL7 was integrated into IL5 to create a more concise item: “I can integrate exercise habits into daily life.”

### 3.2. Participant Demographics

A total of 442 valid responses were collected. The mean age was 21.213 ± 0.785 years (Table 1).

**TABLE 1:** Participant demographic characteristics (*N* = 442).

Characteristic	Category	Frequency	Percentage (%)
Gender	Male	137	30.995
	Female	305	69.005

Year	Freshman	42	9.502
	Sophomore	51	11.538
	Junior	210	47.511
	Senior	115	26.018
	Fifth Year^1^	17	3.846
	Graduate	7	1.584

Major	Humanities	41	9.276
	Science/Tech	80	18.1
	Business/Econ	28	6.335
	Medical/Agri	269	60.86
	Arts/PE	18	4.072
	Military	6	1.357

*Note:* The superscript “1” indicates five‐year undergraduate programs (e.g., Medicine). Some programs in China have a duration of 5 years.

### 3.3. Item Analysis

Item analysis was conducted to evaluate the discriminative power, internal consistency, and structural validity of the preliminary items. The CR method was applied by dividing the total scores into high‐ and low‐score groups (each group comprising the top and bottom 27%, *n* = 119 per group). Independent samples *t*‐tests indicated that all items exhibited strong discriminative power, with CR values ranging from 6.74 to 31.90 (all *p* < 0.001), substantially exceeding the minimum threshold of 3.0. Internal consistency for each item was further assessed. Item–total correlations ranged from 0.259 to 0.765. Correlations for most items exceeded the recommended threshold of 0.40, although several items within the CL dimension fell below this criterion, indicating relatively weaker internal consistency for this subscale. This finding was further supported by the stability of Cronbach’s α when individual items were deleted. The overall scale reliability was high (*α* = 0.892), and removing any single item resulted in negligible changes in α (ranging from −0.0157 to +0.0049), confirming that no item would meaningfully enhance the scale’s reliability if removed. Subsequently, EFA was performed to examine the underlying factor structure. All items loaded strongly onto their hypothesized factors, with factor loadings ranging from 0.591 to 0.927, all exceeding the minimum criterion of 0.40. This indicated robust relationships between the items and their respective dimensions. As all 15 items satisfied the retention criteria (i.e., did not meet two or more deletion criteria), they were retained in the final scale, which comprised the three predefined dimensions. Table 2 shows the detailed results of the item analysis.

**TABLE 2:** Item analysis results for the PA‐HLS.

Item	CR value	Item‐total corr.	α if deleted	Δα	Max factor loading	Decision
FL1: I understand exercise terminology (e.g., “aerobic exercise,” “target heart rate,” “core muscles”)	18.48^∗∗^	0.617	0.883	−0.009	0.591	Retain
FL2: I recognize danger signals during exercise (e.g., joint stabbing pain, persistent dizziness, difficulty in breathing)	19.71^∗∗^	0.632	0.883	−0.009	0.681	Retain
FL3: I can access exercise‐related knowledge from authoritative sources	21.01^∗∗^	0.693	0.881	−0.011	0.596	Retain
FL4: I understand basic nutritional principles (e.g., protein intake) for different exercise goals (muscle gain/fat loss/endurance)	17.68^∗∗^	0.586	0.885	−0.007	0.814	Retain
IL1: I consistently warm up before exercise and cool down afterward	24.23^∗∗^	0.717	0.880	−0.013	0.749	Retain
IL2: When acute injuries occur during exercise (e.g., sprain), I can apply the “RICE principle” (Rest/Ice/Compression/Elevation)	29.91^∗∗^	0.745	0.877	−0.015	0.876	Retain
IL3: I adjust the day’s exercise intensity based on physical state (fatigue level, sleep quality)	31.90^∗∗^	0.749	0.877	−0.016	0.927	Retain
IL4: I proactively learn and rectify incorrect exercise postures (e.g., knees caving in during squats, improper arm swing during running)	30.16^∗∗^	0.764	0.876	−0.016	0.902	Retain
IL5: I integrate exercise habits into daily life (e.g., choosing walking/cycling for commuting, fragmented exercise)	30.54^∗∗^	0.765	0.877	−0.016	0.845	Retain
IL6: I adjust hydration and electrolyte replenishment based on exercise intensity (e.g., sodium‐containing drinks for prolonged exercise)	19.81^∗∗^	0.661	0.881	−0.011	0.758	Retain
CL1: I can distinguish scientific information from exaggerated claims in fitness bloggers or supplement ads	8.73^∗∗^	0.379	0.892	−0.000	0.769	Retain
CL2: I can identify the pseudoscientific nature of claims like “spot reduction” or “7‐day abs”	7.43^∗∗^	0.326	0.894	+0.001	0.848	Retain
CL3: I choose exercise methods by considering interest, physique, and economic conditions, not blindly following trends	6.74^∗∗^	0.259	0.897	+0.005	0.820	Retain
CL4: I can evaluate the applicability and limitations of different exercise theories (e.g., HIIT vs. steady‐state cardio)	8.23^∗∗^	0.300	0.897	+0.005	0.779	Retain
CL5: I believe the core value of scientific exercise for long‐term health outweighs pursuing short‐term physical appearance changes	7.09^∗∗^	0.287	0.897	+0.005	0.818	Retain

*Note:* (1) Factor loadings are absolute values; (2) overall scale *α* = 0.8925.

^∗∗^ *p* < 0.001.

### 3.4. Validity Analysis

#### 3.4.1. Content Validity

Both I‐CVI and S‐CVI achieved perfect scores of 1.0, indicating unanimous expert agreement on the relevance and appropriateness of all items.

#### 3.4.2. Construct Validity

Construct validity was assessed using both EFA and CFA. The total sample of 442 participants was randomly divided into two independent subsamples: one (*n* = 242) for EFA to identify the underlying factor structure, and the other (*n* = 200) for CFA to validate the derived structure.

##### 3.4.2.1. EFA

An EFA was conducted on the 15‐item scale using the first subsample. The data were highly suitable for factor analysis, as indicated by a KMO value of 0.916 and a significant Bartlett’s test of sphericity (*p* < 0.001). PCA with varimax rotation yielded a clear three‐factor solution, accounting for 77.125% of the total variance. The number of factors was determined using both the eigenvalue‐greater‐than‐one criterion and the inflection point in the scree plot (Appendix Figure A2), which strongly supported the three‐factor model based on Nutbeam’s theoretical framework. The factors were labeled as CL (items CL1–CL5), IL (items IL1–IL6), and FL (items FL1–FL4). All items exhibited strong loadings on their assigned factors, ranging from 0.56 to 0.90 (Appendix Figure A3), with no notable cross‐loadings, confirming a clear and interpretable factor structure.

##### 3.4.2.2. CFA

To validate the three‐factor structure identified in the EFA, a CFA was performed on the second subsample (*n* = 200). The model showed acceptable to good fit indices: *χ* ^2^/df = 2.132, RMSEA = 0.089, CFI = 0.944, and GFI = 0.912, all meeting established criteria for model adequacy. These results provide robust empirical support for the hypothesized three‐dimensional structure of the PA‐HLS. Standardized factor loadings are displayed in Appendix Figure A4.

#### 3.4.3. Discriminant Validity

Discriminant validity was evaluated by comparing interfactor correlations with the square roots of the AVE for each latent construct. As shown in Table 3, correlations among the factors were low to moderate, with a relatively strong correlation observed between functional and interactive health literacy (*r* = 0.719). Importantly, the square root of the AVE for each factor (range: 0.785–0.884) exceeded its correlations with other factors, satisfying the criterion for discriminant validity.

**TABLE 3:** Discriminant validity analysis results (*n* = 200).

Dimension	FL	IL	CL
FL	**0.823**		
IL	0.719^∗∗∗^	**0.884**	
CL	−0.052	0.049	**0.785**

*Note:* Diagonal (bold)​ values are the square roots of the AVE; off‐diagonal values are Pearson’s correlation coefficients between dimensions.

^∗∗∗^ *p* < 0.001.

### 3.5. Reliability Analysis

Internal consistency reliability was assessed using Cronbach’s α, split‐half reliability, and McDonald’s ω. The overall Cronbach’s α coefficient was 0.892, indicating good internal consistency. For the subscales, Cronbach’s α values were 0.881 for FL, 0.951 for IL, and 0.898 for CL. The split‐half reliability coefficient (adjusted for unequal length) was 0.766 for the total scale, with corresponding values of 0.871, 0.927, and 0.868 for FL, IL, and CL, respectively. McDonald’s ω coefficients further supported the internal consistency of the PA‐HLS. The ω values were 0.8956 for FL, 0.9527 for IL, and 0.8889 for CL. The omega coefficient for the total scale was 0.8687, indicating good overall reliability.

### 3.6. Results of Rasch Model Analysis Based on Item Response Theory

#### 3.6.1. Unidimensionality Test

PCA of residuals was conducted to evaluate the unidimensionality assumption, a prerequisite for Rasch model analysis. For the full scale, the first eigenvalue was 6.872, confirming the intended multidimensional structure. At the subscale level, the FL subscale satisfied the unidimensionality criterion, with the first eigenvalue of 2.971. For the IL and CL subscales, the first eigenvalues were 4.838 and 3.601, respectively. The ratios of the second to first eigenvalues for these subscales were 0.090 (IL) and 0.143 (CL), indicating essential unidimensionality.

#### 3.6.2. Item Fit

Infit and outfit mean‐square (MNSQ) values for the majority of items fell within the acceptable range of 0.7–1.3, with corresponding standardized residuals (ZSTD) within ±2.0, indicating adequate model fit. Three items (FL4, IL6, and CL2) showed minor deviations from these criteria. However, ZSTD statistics are sensitive to sample size and may be inflated into larger samples. In contrast, the corresponding infit and outfit MNSQ values for these items remained within or close to the acceptable range, suggesting that the extent of misfit was minimal. As MNSQ statistics are generally considered more informative indicators of item fit, these deviations were deemed acceptable and did not substantially compromise the overall model fit or the measurement quality of the scale. Point–measure correlation coefficients for all items exceeded 0.80, further supporting their discriminative power (Table 4).

**TABLE 4:** Rasch model item statistics for the health literacy scale.

Item	Measure	S.E.	Infit MNSQ	Infit ZSTD	Outfit MNSQ	Outfit ZSTD	PTMEA CORR
FL1	1.30	0.12	1.14	1.6	1.19	2.0	0.87
FL2	−0.60	0.13	0.86	−1.7	0.79	−2.2	0.86
FL3	−0.32	0.13	1.15	1.7	1.12	1.2	0.83
FL4	−0.38	0.13	0.75	−3.2	0.69	−3.4	0.88
IL1	−2.43	0.13	1.06	0.9	0.99	0.0	0.84
IL2	−0.20	0.13	0.85	−1.7	0.78	−2.1	0.92
IL3	1.14	0.12	0.73	−4.1	0.65	−4.2	0.94
IL4	0.19	0.13	0.88	−1.4	0.78	−2.3	0.92
IL5	−0.88	0.13	0.88	−1.4	0.87	−1.1	0.91
IL6	2.18	0.12	1.34	4.8	1.44	4.0	0.85
CL1	−1.09	0.13	1.02	0.2	1.10	0.9	0.80
CL2	−0.69	0.12	0.78	−2.5	0.76	−2.5	0.85
CL3	−0.15	0.12	1.04	0.5	0.96	−0.3	0.84
CL4	1.17	0.11	1.10	1.2	1.14	1.4	0.85
CL5	0.76	0.11	0.89	−1.3	0.84	−1.7	0.85

*Note:* Measure: item difficulty (in logits); Infit MNSQ: information‐weighted mean square fit statistic; outfit MNSQ: outlier‐sensitive mean square fit statistic; ZSTD: standardized fit statistic; PTMEA CORR: point‐measure correlation.

Abbreviation: S.E., standard error.

#### 3.6.3. Reliability and Separation Analysis

The person reliability for the overall scale was 0.84, and the item reliability was 0.97. The reliability indices for the subscales were 0.77 (FL), 0.92 (IL), and 0.82 (CL). Both person and item separation indices for the overall scale and subscales exceeded 2.0, indicating adequate measurement precision (Table 5). The ICCs for all items closely followed the model‐predicted trajectories (Figure 1(a)). The distribution of person abilities and item difficulties on the Wright map (Figure 1(b)) showed substantial overlap between approximately −1.0 and 1.5 logits. However, item density decreased at the higher end of the ability continuum (above approximately 1.8 logits), suggesting limited precision for individuals with high ability levels.

**TABLE 5:** Rasch model fit and reliability statistics for the overall scale and subscales.

Scale/subscale	Infit MNSQ	Outfit MNSQ	Person measures		Item measures	
			Separation	Reliability	Separation	Reliability
Overall Scale	1.01 ± 0.62	1.01 ± 0.63	2.33	0.84	6.04	0.97
FL	0.93 ± 1.03	0.95 ± 1.09	1.85	0.77	5.62	0.97
IL	0.95 ± 0.93	0.92 ± 1.08	3.30	0.92	11.06	0.99
CL	0.95 ± 1.07	0.96 ± 1.11	2.13	0.82	7.07	0.98

*Note:* Infit MNSQ = information‐weighted mean square fit statistic; outfit MNSQ = outlier‐sensitive mean square fit statistic. The person separation index reflects the number of statistically distinct ability strata that the scale can reliably differentiate. The item separation index indicates the scale’s capacity to establish a well‐defined and distinct hierarchy of item difficulties. Reliability is analogous to internal consistency, representing the precision and stability of measurement.

FIGURE 1Item characteristic curves (ICCs) and Wright map (person‐item map) of the health literacy scale from Rasch analysis. (a) ICCs illustrate the probability of a positive response across varying levels of person ability (in logits). The solid lines represent the model‐expected curves, and the dots represent the empirically observed response proportions. (b) The Wright map presents the distribution of person ability (left) and item difficulty (right) on a shared logit scale. E‐ICC: expected score ICC.

**Figure:**
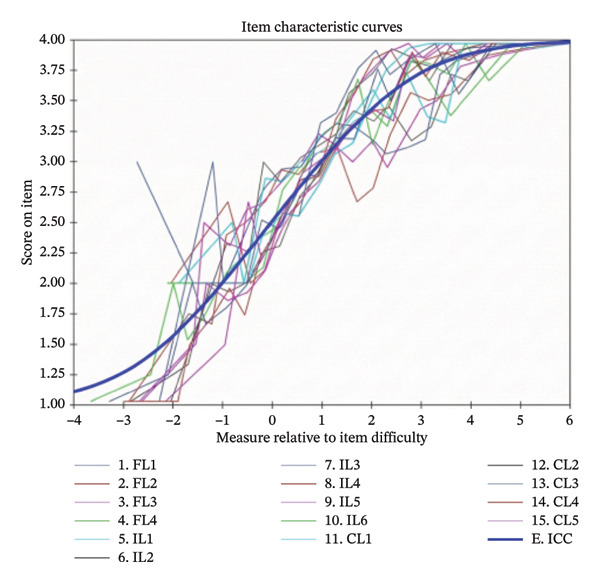
(a)

**Figure:**
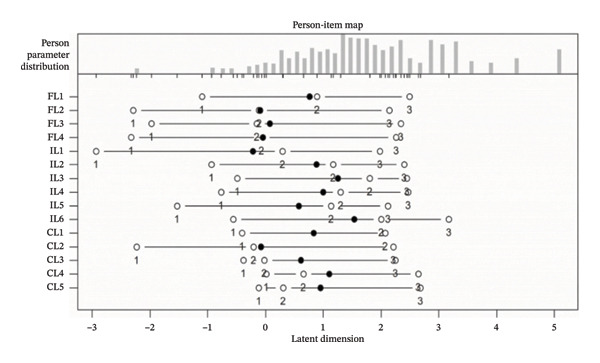
(b)

## 4. Discussion

### 4.1. Innovation in Scale Development

This study developed the PA‐HLS based on Don Nutbeam’s hierarchical model of health literacy, addressing the growing need for domain‐specific health literacy assessment [29]. By situating PA within a health literacy framework, the PA‐HLS extends existing conceptualizations of PL, which have predominantly focused on physical competence, motivation, and affective attributes. The distinctive characteristics of PA‐related information further justify the development of a domain‐specific instrument. Compared with other health domains, such as sleep or mental health, PA information is more frequently embedded in commercial contexts, closely related to body image outcomes, and characterized by a high prevalence of misinformation and pseudoscientific claims. Within this complex information environment, individuals must not only acquire and apply knowledge but also critically evaluate the credibility, applicability, and potential bias of information sources. This highlights the importance of the CL dimension in the PA‐HLS, which captures individuals’ capacity to make informed decisions when confronted with complex and potentially misleading PA‐related information.

A key innovation of the PA‐HLS is its explicit framing of PA as a health information processing task. Rather than measuring perceived physical capabilities or levels of engagement, the scale operationalizes FL, IL, and CL competencies specific to PA. This approach emphasizes individuals’ abilities to access, interpret, apply, and critically evaluate PA‐related information, which is increasingly relevant in contemporary digital environments characterized by fragmented information sources and widespread misinformation.

Conceptually, the PA‐HLS adapts Nutbeam’s three‐level model to the context of PA, reflecting a progression from basic knowledge acquisition to behavioral application and informed decision‐making. FL encompasses foundational knowledge required for safe participation in PA; IL involves applying knowledge and regulating behavior in daily exercise contexts, and CL includes higher‐order evaluative skills necessary to distinguish scientific evidence from pseudoscientific or commercially motivated claims. This structure aligns with the cognitive and behavioral challenges faced by college students navigating diverse PA information across online and offline platforms.

Compared to existing PL instruments, the PA‐HLS addresses a conceptual gap by prioritizing health information competence over physical performance or self‐perceived ability. Tools such as the Physical Literacy in Children Questionnaire (PL‐C Quest) [30], the Perceived Physical Literacy Instrument and its adaptations [31, 32], and the Physical Literacy in Adults Scale (PLAS) [33] offer valuable assessments of the physical, psychological, and social dimensions of PL but do not explicitly measure PA‐specific health literacy skills. In contrast, the PA‐HLS provides a theoretically grounded instrument designed to assess competencies directly linked to informed PA decision‐making, thereby complementing rather than duplicating existing PL measures (Appendix Table A1).

### 4.2. Scientific Rigor of the Scale

The findings of this study demonstrate that the PA‐HLS exhibits strong psychometric properties and meets contemporary standards for scale development and validation. Unlike many existing PL and health literacy instruments, which rely primarily on EFA and internal consistency estimates, this study employed a comprehensive validation strategy integrating classical test theory and item response theory.

Both EFA and CFA supported a stable three‐factor structure consistent with Nutbeam’s theoretical framework. The use of independent subsamples for EFA and CFA provided robust evidence for structural validity of the scale, reducing the risk of overfitting and enhancing confidence in the generalizability of the factor structure. Internal consistency indices, including Cronbach’s α and McDonald’s ω, indicated high reliability for the total scale and all subscales, confirming the internal coherence of the PA‐HLS [21].

Importantly, the inclusion of Rasch model analysis represents a methodological advancement beyond conventional validation methods typically used in PL research [27]. Rasch analysis enabled item‐level evaluation of fit, reliability, and separation, offering additional evidence for the measurement quality and unidimensionality of each subscale. Such item response theory–based validation remains relatively rare in the development of PL‐related instruments for college populations, thereby strengthening the interpretability and robustness of the PA‐HLS.

The IL dimension accounted for the largest proportion of explained variance in the EFA. This finding likely reflects the characteristics of contemporary college students, whose engagement with PA is often mediated by interactive information‐seeking, self‐regulation, and behavioral adaptation, particularly within digital and social media environments. In such contexts, the ability to apply and adapt health information may be more salient than foundational functional knowledge alone. In addition, a relatively strong correlation was observed between the FL and IL dimensions (*r* = 0.719). Although the Fornell–Larcker criterion supported discriminant validity, this high correlation suggests a degree of conceptual overlap between the two constructs.

This finding is theoretically plausible, as both FL and IL involve core processes of acquiring, understanding, and applying health‐related information. In the context of PA, individuals with strong functional knowledge, such as an understanding of exercise principles, are more likely to translate this knowledge into practice, resulting in a close association between the two dimensions. From a measurement perspective, this pattern may reflect the progressive nature of Don Nutbeam’s hierarchical model, in which IL builds upon FL. However, the relatively high correlation also suggests that the distinction between these constructs may not be entirely clear‐cut in this population. Future research could further examine the discriminant boundaries between FL and IL, for example, by refining item content or exploring alternative factor structures across diverse populations.

Notably, several items within the CL dimension exhibited relatively lower item‐total correlations, indicating comparatively weaker internal consistency in this subscale. This pattern may reflect the inherently complex and multidimensional nature of CL in the context of PA. One possible explanation is that CL involves higher‐order cognitive processes—such as evaluating the credibility of exercise‐related information, identifying pseudoscientific claims, and making informed decisions under conditions of uncertainty—which may vary considerably among college students. Such variability likely contributes to increased response heterogeneity and, consequently, lower interitem correlations. From a measurement standpoint, this pattern suggests that the CL construct may be less cohesive than FL or IL, highlighting a potential limitation of the current scale structure. Nonetheless, the items demonstrated strong factor loadings and satisfactory fit within the Rasch model, supporting their role in the overall construct validity of the scale. They were therefore retained to maintain the theoretical breadth of the CL dimension.

Overall, converging evidence from factor analyses, reliability estimates, discriminant validity testing, and Rasch modeling supports the conclusion that the PA‐HLS is a psychometrically robust and methodologically rigorous instrument for assessing PA‐related health literacy among college students.

### 4.3. Limitations and Future Directions

Several limitations of this study should be acknowledged. First, the sample was drawn from a single cultural context, which may limit the generalizability of the findings to college students in other regions or countries. Future research should validate the PA‐HLS in diverse cultural and educational settings to examine its cross‐cultural applicability and measurement invariance. Second, test–retest reliability was not assessed; therefore, the temporal stability of the PA‐HLS remains unclear. Longitudinal studies are needed to evaluate the stability of the scale over time and its sensitivity to changes following interventions. Third, although content validity was assessed using the CVI, the Content Validity Ratio (CVR) was not calculated, which may slightly limit the strength of content validity evidence. Future studies could incorporate additional quantitative approaches to further strengthen content validation. Finally, the current study relied on self‐reported data, which may be subject to social desirability bias. Future research could combine objective measures of PA behavior or experimental designs to further examine the predictive validity and practical utility of the PA‐HLS.

## 5. Conclusion

The PA‐HLS is a theoretically grounded and psychometrically robust instrument designed to assess PA‐related health literacy among college students. Grounded in Nutbeam’s hierarchical model, the scale captures FL, IL, and CL competencies relevant to PA engagement in contemporary, information‐rich environments. Evidence from both classical test theory and item response theory supports the scale’s strong reliability, clear factor structure, and satisfactory item‐level performance. The PA‐HLS provides a practical tool for evaluating health literacy competencies associated with PA and may help identify specific educational needs among college students. The PA‐HLS may be applied in future research and practice to inform health promotion strategies, guide intervention development, and support the evaluation of PA‐related educational initiatives in university settings.

## Author Contributions

Pei Duan: conceptualization, methodology, funding acquisition, investigation, formal analysis, and writing–original draft.

Xia Song: conceptualization, methodology, writing–original draft, and writing–review and editing.

Xinrui Zhang: investigation and formal analysis.

Liqian Ruan: investigation and formal analysis.

Song Chen: investigation and formal analysis.

## Funding

This work was supported by the Huxin Fund of Jiangsu Key Laboratory of Zoonosis (Grant Number HX2110).

## Conflicts of Interest

The authors declare no conflicts of interest.

## Supporting Information

Additional supporting information can be found online in the Supporting Information section.

## Supporting information


**Supporting Information** Supporting File 1: Appendix Figures and Tables for the PA‐HLS study, including: Figure A1: University Student Physical Health Literacy Hierarchical Model. Figure A2: Factor Analysis Scree Plot. Figure A3: Factor Loading Matrix Heatmap. Figure A4: Confirmatory Factor Analysis Model of the PA‐HLS. Table A1: Existing Physical Literacy/Health Literacy Scales and their characteristics.

## Data Availability

The datasets generated and/or analyzed during the current study are available from the corresponding author upon reasonable request.
