# Supplementary material for: Development and Psychometric Validation of the Physical Activity and Health Literacy Scale (PA‐HLS) for College Students
Source: Nurs Res Pract. 2026 May 13;2026:9980613. doi: 10.1155/nrp/9980613 (PMC13172606; doi:10.1155/nrp/9980613)
Supplement: Supplementary file 1 — Supporting Information Supporting File 1: Appendix Figures and Tables for the PA‐HLS study, including: Figure A1: University Student Physical Health Literacy Hierarchical Model. Figure A2: Factor Analysis Scree Plot. Figure A3: Factor Loading Matrix Heatmap. Figure A4: Confirmatory Factor Analysis Model of the PA‐HLS. Table A1: Existing Physical Literacy/Health Literacy Scales and their characteristics. [file NRP-2026-9980613-s001.docx]

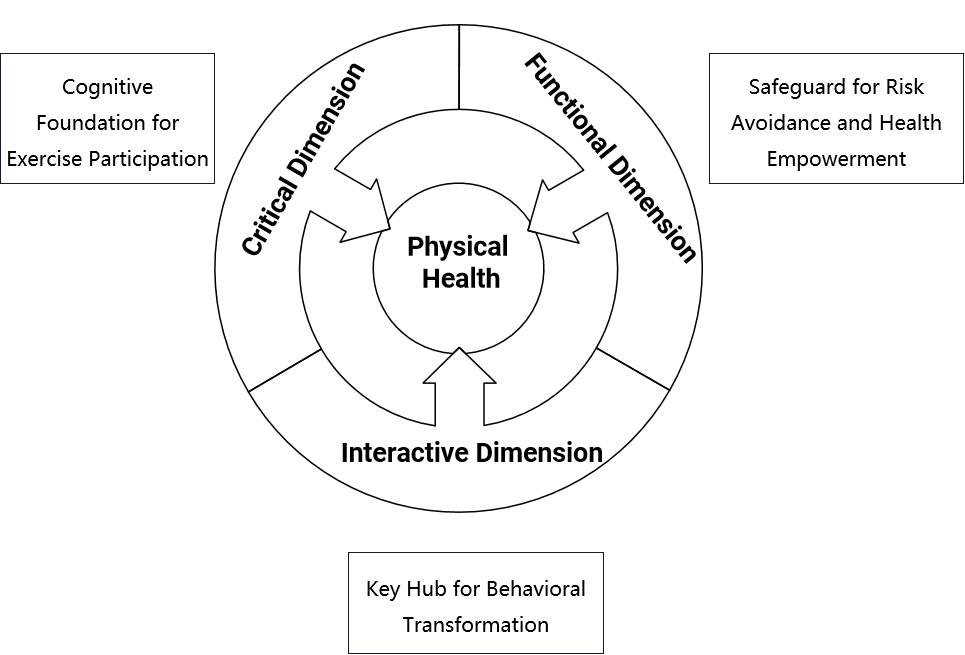


*Appendix Figure A1. University Student Physical Health Literacy Hierarchical Model Adapted from Nutbeam (2000) Health Literacy Framework*


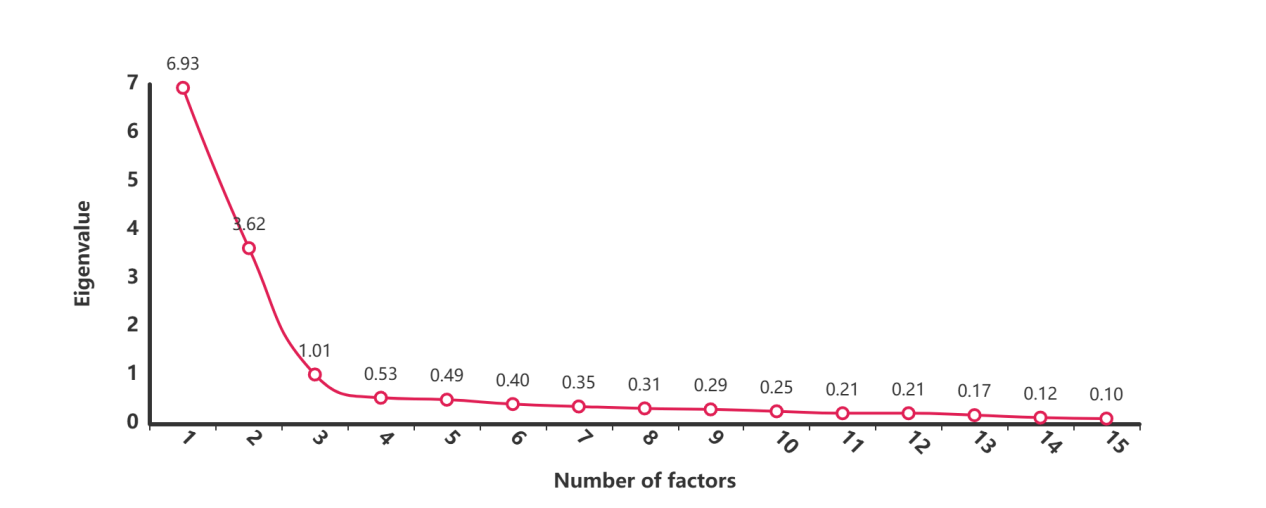


Appendix Figure A2. Factor Analysis Scree Plot. Note: Based on the steep decline in eigenvalues, the curve flattens after the third factor, supporting the extraction of three factors.


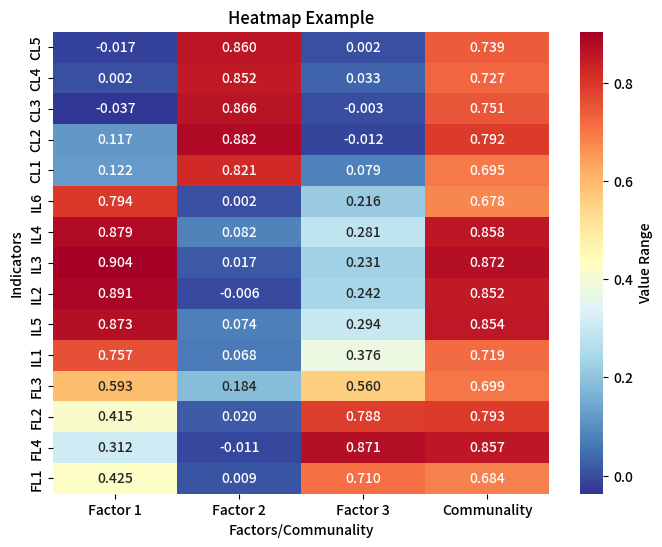


Appendix Figure A3. Factor Loading Matrix Heatmap. Note: Red indicates positive correlation, blue indicates negative correlation; darker color indicates larger loading magnitude.


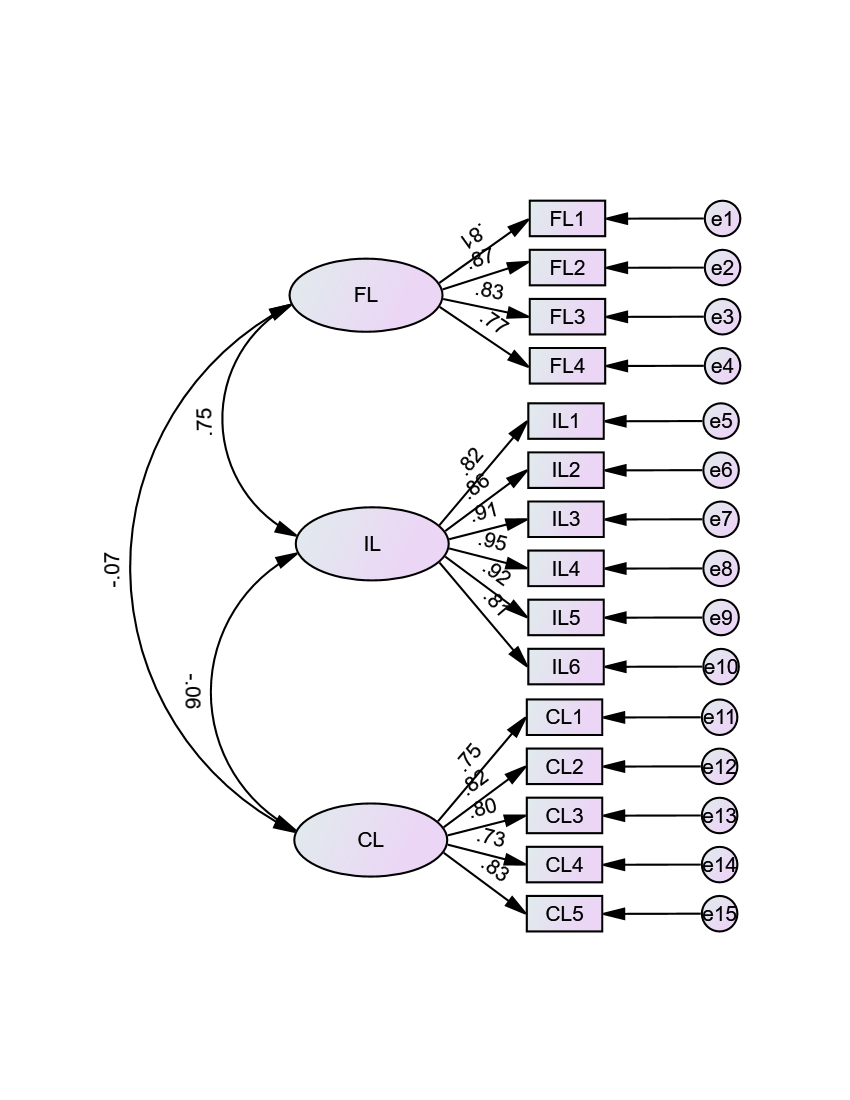


*Appendix Figure A4. Confirmatory Factor Analysis Model of the PALS(Standardized Estimates).*

Appendix Table A1. Existing Physical Literacy/Health Literacy Scales

| **Name** | **Target Group** | **Characteristics** | **Core Dimensions and Reliability** |
| --- | --- | --- | --- |
| **Physical Literacy in Children Questionnaire, PL-C Quest^[1]^** | **Children aged 8-11** | **Focuses on children** | **Core Dimensions：Physical competence / Psychological & affective / Cognitive understanding / Environmental interaction.ICC=0.94，95% CI：0.90-0.96，p<0.001；Pα=0.90** |
| **Perceived Physical Literacy Instrument for Teachers, PPLI-T^[2]^** | **PE Teachers (esp. in HK primary/secondary)** | **Focuses on teachers' subjective perceptions of students' PL development** | **Cronbach’sα=0.82 Factor 1 (Knowledge and Understanding): Cronbach’s α = 0.73**  **Factor 2 (Sense of Self and Self-Confidence): Cronbach’s α = 0.76**  **Factor 3 (Self-Expression and Communication with Others): Cronbach’s α = 0.76** |
| **Simplified Chinese Version of Perceived PL Instrument for Undergrads^[3]^** | **Undergraduates** | **Measures perceived PL in Chinese undergraduates** | **Cronbach’sα=0.86 Factor 1 (Confidence and Physical Competence): Cronbach’s α = 0.80**  **Factor 2 (Motivation): Cronbach’s α = 0.68**  **Factor 3 (Interaction with the Environment): Cronbach’s α = 0.90** |
| **Physical Literacy in Adults Scale, PLAS^[4]^** | **Adults aged 18-75** | **Fills the gap in adult PL assessment, particularly focusing on middle-aged and older adults** | **Cronbach’sα=0.87**  **Motivation、Confidence、**  **Social、Strength、Knowledge：Cronbach’sα=0.70-0.90** |

Notes: 1. ICC (Intraclass Correlation Coefficient): Used to assess the test-retest reliability (stability between two measurements) of the scale. The criteria are: ≥0.90 = excellent, 0.75-0.89 = good, 0.50-0.74 = moderate; 2. Pα (Polychoric Ordinal Alpha): Applied to evaluate the internal consistency reliability of ordinal data. The criteria are: ≥0.90 = excellent, 0.70-0.89 = good.

***References***

[1]Richard Tyler,Caitlin Miller,Lisa M. Barnett, et al. Validity and reliability of the Physical Literacy in Children Questionnaire (PL-C Quest) for primary school children aged 8–11 years[J]. Journal of Science and Medicine in Sport, 2025, 28: 483-490.

[2]Raymond Kim Wai Sum,Amy Sau Ching Ha,Chih Fu Cheng, et al. Construction and Validation of a Perceived Physical Literacy Instrument for Physical Education Teachers[J]. Plos One, 2016, 11: e0155610.

[3]Rui-Si Ma,Raymond K.W. Sum,Yu- Na Hu, et al. Assessing factor structure of the simplified Chinese version of Perceived Physical Literacy Instrument for undergraduates in Mainland China[J]. Journal of Exercise Science & Fitness, 2020, 18: 68-73.

[4]Annaleise Naylor,Andrew Flood,Lisa M. Barnett, et al. Development of the Physical Literacy in Adults Scale (PLAS)[J]. Journal of Sports Sciences, 2024, 42: 1099-1111.
